# Coordinated Actions of MicroRNAs with other Epigenetic Factors Regulate Skeletal Muscle Development and Adaptation

**DOI:** 10.3390/ijms18040840

**Published:** 2017-04-15

**Authors:** Marzia Bianchi, Alessandra Renzini, Sergio Adamo, Viviana Moresi

**Affiliations:** 1 DAHFMO Unit of Histology and Medical Embryology, Interuniversity Institute of Myology, Sapienza University of Rome, Via Antonio Scarpa 14, 00161 Rome, Italy; marziabi@inwind.it (M.B.); alessandra_renzini@yahoo.it (A.R.); sergio.adamo@uniroma1.it (S.A.); 2 Laboratory of Cardiovascular Endocrinology, IRCCS San Raffaele Pisana, 00166 Rome, Italy

**Keywords:** miRNA action with epigenetic factors, miRNAs and DNA methylation, miRNAs and HATs/HDACs, miRNAs and PRC2/Ezh2/Prmts

## Abstract

Epigenetics plays a pivotal role in regulating gene expression in development, in response to cellular stress or in disease states, in virtually all cell types. MicroRNAs (miRNAs) are short, non-coding RNA molecules that mediate RNA silencing and regulate gene expression. miRNAs were discovered in 1993 and have been extensively studied ever since. They can be expressed in a tissue-specific manner and play a crucial role in tissue development and many biological processes. miRNAs are responsible for changes in the cell epigenome because of their ability to modulate gene expression post-transcriptionally. Recently, numerous studies have shown that miRNAs and other epigenetic factors can regulate each other or cooperate in regulating several biological processes. On the one hand, the expression of some miRNAs is silenced by DNA methylation, and histone modifications have been demonstrated to modulate miRNA expression in many cell types or disease states. On the other hand, miRNAs can directly target epigenetic factors, such as DNA methyltransferases or histone deacetylases, thus regulating chromatin structure. Moreover, several studies have reported coordinated actions between miRNAs and other epigenetic mechanisms to reinforce the regulation of gene expression. This paper reviews multiple interactions between miRNAs and epigenetic factors in skeletal muscle development and in response to stimuli or disease.

## 1. Introduction

Numerous studies have demonstrated the importance of epigenetic mechanisms in regulating the development and the responses to external stimuli in virtually any cell type. By remodeling the chromatin structure or gene expression, epigenetics cooperates with transcription factors and the translational machinery in fine-tuning gene expression. Cells are often paused in a primed state through epigenetic mechanisms, and their activation is triggered by external stimuli. For instance, quiescent skeletal muscle stem cells are primed for myogenesis, as they display permissive marks for gene transcription, coupled with a lack of repressive chromatin marks in several genes [1]. Indeed, by modulating the last steps of gene expression, cells can rapidly respond to stress or demands. At the same time, epigenetics explains some phenotypes caused by bad habits. An example is the appearance of metabolic dysfunction, which can be reversed by exercise, in the offspring of mice fed with a high-fat diet, due to epigenetic modifications in the promoter of one of the metabolic master regulators, peroxisome proliferator-activated receptor γ coactivator-1α [2]. In this article, we review miRNAs, among the different epigenetic mechanisms, focusing on the interplay between miRNAs and other epigenetic players in skeletal muscle development and in response to pathological conditions.

### 1.1. MicroRNAs

miRNAs belong to the category of small, regulatory, non-coding RNA molecules, which also include small interfering RNAs and repeat-associated siRNAs. miRNAs are mostly located within the cell, although, recently some of them have also been found extracellularly, in biological fluids and cell culture media [3]. The mechanisms of miRNA selective release are largely unknown, as well as their function in distant cell-to-cell communication. However, it is clear that circulating miRNAs adopt some protective mechanisms since they are resistant to high RNase activity of the extracellular environment and some of them can be associated with patho-physiological conditions [4,5]. miRNAs can be found in clusters and, therefore, transcribed as polycistronic primary transcripts, or as independent units, including intergenic regions, exon sequences of non-coding transcripts, or intronic sequences of either protein-coding or non-coding transcripts [6,7]. If miRNAs are located in an intronic region and have the same direction as the host genes, they are generally transcribed simultaneously and excised by the splicing machinery [8]. Certainly, transcribing intronic miRNAs may represent an efficient mechanism for a protein-coding gene to regulate the expression of other proteins [9].

miRNAs are estimated to be responsible for the regulation of about 60% of human genes [10]. A single miRNA can target dozens of mRNAs, whereas individual mRNAs can be targeted by multiple miRNAs, allowing for additional levels of complexity in gene expression regulation. miRNAs often serve to fine-tune gene expression, but they have also been reported as being able to switch gene expression on and off [11]. The miRNA region, which encompasses nucleotides 2–8 at the 5′ end, defined as the seed region, is crucial for target recognition. Generally, the seed region completely pairs at the 3′ untranslated region (UTR) of the target mRNAs [12], although miRNAs can also bind to the 5′ UTRs or to the coding regions of their target [13,14,15,16,17,18,19]. Moreover, some miRNA-target interactions do not occur through the seed regions, but via non-canonical sites [20,21].

miRNAs regulate gene transcription via two main mechanisms which vary according to the degree of complementarity between a miRNA and its target: high complementarity leads to mRNA cleavage of the target through the RNA-induced silencing complex (RISC) [22], while low complementarity induces translational inhibition of the mRNA target [23,24]. In animal cells, miRNAs predominantly regulate gene expression via translational inhibition, either by interfering with the ribosome assembly or by inducing its early dissociation [25,26,27,28,29]. In addition, miRNAs with partial complementarity to their target mRNAs promote mRNA degradation via deadenylation, followed by decapping and the ensuing exonucleolytic digestion [29,30,31].

It is difficult to establish how many miRNAs are encoded by the human genome, because of their small size and nature (i.e., noncoding) and the different criteria used for miRNA annotation by investigators. Several studies claim to have found thousands of mature miRNAs [32,33,34], while others argue that in miRBase, the online repository for miRNAs, there are many false positives, i.e., sequences that are not derived from authentic miRNA genes [35]. One of the most recent and comprehensive analyses of human miRNA abundance in different tissues has profiled 1997 different mature miRNAs for 61 tissues, by using a microarray platform for miRNA expression [34]. The vast majority of miRNAs (>80%) showed an average abundance level throughout the tissues, about 10% showed a high tissue-specific expression, indicating that miRNA expression is more tissue specific compared to mRNA expression, while only one miRNA proved to be ubiquitously expressed. In addition, the high expression of miR-1-3p, miR-133a-3p, miR-133b, and miR-206 in myocard and muscles was confirmed.

### 1.2. DNA Methylation

DNA methylation is an epigenetic mechanism of gene silencing achieved through the addition of methyl groups to cytosines within CpG dinucleotides, frequently present in clusters in the genome [36]. DNA methylation, per se, does not imply transcriptional silencing; it acts as a docking site for the recruitment of other proteins and chromatin remodeling factors to repress gene expression [36]. During embryonic development, DNA methylation determines the maintenance of mono-allelic silencing in genomic imprinting and X chromosome inactivation [37]. Indeed, the methylation status differs from expressed and silenced genes and a loss of DNA methylation results in a loss of imprinting [38]. During embryogenesis, few genes change their methylation status, except for those expressed in the germline [39]. DNA methylation appears to be dispensable in undifferentiated cells, whereas it is absolutely required in differentiated cells, as genetic deletion of DNA methyltransferase results in embryonic or post-partum lethality [40]. Moreover, DNA methylation plays a pivotal role in regulating gene expression in response to external stimuli, as in the expression of metabolic or developmental genes in the offspring of pregnant mice exposed to different insults [2,41]. Further studies have delineated the biological impact of DNA methylation in cancer by silencing DNA methyltransferases, or by treating cells with DNA demethylating agents [40]. For instance, hyper-methylation has been proposed as a prognostic biomarker in acute lymphoblastic leukemia, since it is associated with higher mortality rates [42].

### 1.3. Histone Modifications

Histones are not just structural proteins responsible for packing chromatin; they are active regulators of gene expression and can undergo several post-translational and chemical modifications that alter their physical interaction and spatial distribution. Histone modifications, which include acetylation, methylation, phosphorylation, deimination, ubiquitylation, sumoylation, and ADP ribosylation, affect the chromatin structure and create affinities for chromatin-associated proteins, thereby modulating gene expression [43]. Histone modifications are responsible for the variation in the expression of genes involved in diverse signaling pathways, regulating many cellular processes, such as proliferation, differentiation, or repair [44]. Alteration in histone modifications are often associated with diseases and are set to cause, or participate in, the onset and/or progression of pathological states [45]. Specific combinations of histone modifications occurring on the same histone tail, or on another tail, confer the overall expression status of a DNA region, a theory known as the “histone code” [46]. The “histone code” is deciphered by specific proteins, able to bind to histone modifications, and consequently remodel the chromatin structure.

The acetylation of lysine residues within histone tails, catalyzed by histone acetyltransferases (HATs), neutralizes histone positive charges, hence, facilitating chromatin relaxation and increasing the accessibility of transcription factors to their target genes [47]. The action of HAT is counteracted by histone deacetylases (HDACs), which allow chromatin compaction and the repression of gene transcription [48]. Generally, histone hypoacetylation and hypermethylation characterize silenced DNA sequences, as in the inactive X chromosome in females or imprinted genes. Polycomb Repressive Complex 2 (PRC2) drives trimethylation of histone 3 lysine 27 (H3K27me3), establishing gene silencing at developmentally-regulated loci. Conversely, trithorax group proteins, which mediate the trimethylation of histone 3 lysine 4 (H3K4me3), together with the histone lysine-specific demethylases, antagonize PRC2 repressive activity and allow gene expression in specific cell types [49]. In muscle stem cells, the absence of the repressive mark H3K27me3 across the genome and the concomitant existence of H3K4me3 at the transcription start sites maintain chromatin in a primed state, allowing cells to quickly respond to external stimuli [1]. Histone phosphorylation affects the “histone code” by influencing the hierarchy of subsequent histone posttranslational modifications [50]. Indeed, not only does histone phosphorylation dictate the precise spatiotemporal histone phosphorylation patterns of adjacent serines or threonines, but it also influences the acetylation status and might affect the readout of stable methylation marks at the neighboring lysine residues. Hence, it regulates the binding of effector proteins to histones, during the cell cycle or in response to stress activation [51,52,53]. Citrullination is the post-translational conversion of a histone arginine residue to the non-coded amino acid citrulline [54], thus affecting chromatin compaction and cellular processes. For instance, it antagonizes arginine methylation during transcriptional activation [55], it regulates DNA damage response upon cellular stress [56], and it controls reprogramming efficiency by activating the expression of key stem-cell genes during embryogenesis [57]. Moreover, histone ubiquitination influences other posttranslational modifications, such as histone methylation [58], thus affecting gene transcription [59]. Similarly, histone sumoylation cooperates with HDACs and mediates the repression of gene transcription [60]. Instead, histone ADP-ribosylation directly destabilizes nucleosomes, leading to the activation of gene transcription [61] and influencing DNA repair and cell replication [62]. 

### 1.4. MiRNAs and Epigenetics

miRNAs contribute to and are part of the epigenetic regulation of gene expression in at least three different ways: first, the expression of miRNAs is regulated by multiple epigenetic mechanisms; secondly, miRNAs can repress the expression of epigenetic factors; and thirdly, miRNAs and epigenetic factors can cooperate to modulate common targets.

As for the coding regions, epigenetics also plays an important role in regulating miRNA expression. About half of the miRNA genes encompass CpG islands. Some of them are regulated by DNA methylation in tumors and in a cancer-specific fashion, such as miR-31 in breast cancer [63], or miR-124a in colon cancer [64].

Several histone modifications have been involved in the regulation of miRNA expression in cancer or during development [65]. Moreover, DNA methylation and histone modifications often cooperate to regulate miRNA expression, as highlighted by experiments with HDAC inhibitors together with DNA demethylating agents or DNA methylation inhibitors [65]. Interestingly, a feedback regulation exists, since, in turn, some miRNAs are able to regulate the expression of epigenetic factors. For example, the expression of DNA methyl transferases is repressed by the miR-29 or the miR-148 families [66,67]; histone-modifying enzymes are regulated by miR-449a, miR-101, and miR-137 [68,69,70]. These feedback regulations create a complex network between miRNAs and the epigenetic machinery, which strengthens the epigenetic regulation of gene expression. 

In this review, we discuss how miRNAs are regulated by, actively repress, or cooperate with epigenetic factors in skeletal muscle.

## 2. MicroRNAs and DNA Methylation

**Figure 1:**
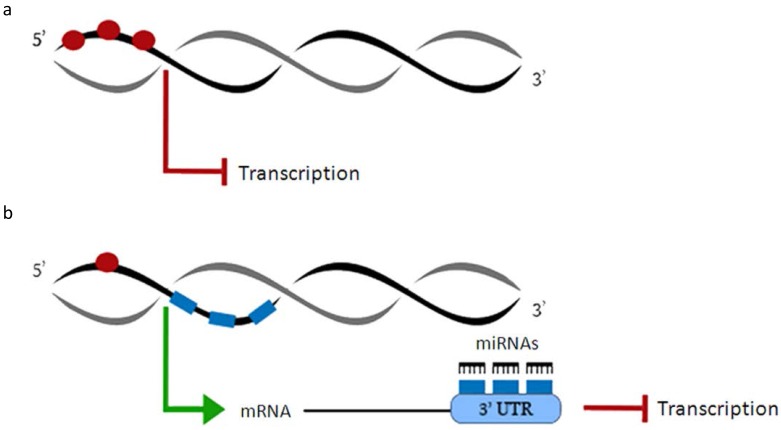
Functional complementation between methylation of promoter regions and miRNA post-transcriptional regulation. DNA methylation of promoter regions (**a**) and miRNA regulation (**b**) are usually mutually exclusive. Red dots: promoter methylated sites; blue rectangles: miRNA binding sites.

DNA methylation and post-transcriptional gene silencing by miRNAs are two important epigenetic mechanisms of skeletal muscle development and adaptation to diseases. There is evidence that DNA methylation and miRNAs cooperate in the suppression of gene expression and protein translation of common targets [71]. However, how these two major mechanisms combine to influence skeletal muscle homeostasis and functioning remains unclear. Taking advantage of recent deep-sequencing technologies, papers in which DNA methylation profile is compared with the full spectrum of expressed miRNAs (miRNAome) and the consequent transcriptome network have started to appear. The first-in-human study, where a coordinated action between promoter methylation and miRNAs was suggested, was based on some considerations: (1) DNA methylation acts on the 5′ promoter region of a gene; (2) gene transcription typically depends on demethylation of the promoter regions; and (3) miRNAs target the 3′ UTR to suppress gene expression. The authors hypothesized the existence of a functional complementation between the methylation of promoter regions and the post-transcriptional regulation guaranteed by miRNAs [71]. By systematic genome-wide examination, it was shown that genes with a low methylation level have more miRNA binding sites on their mRNA 3′ UTRs, while genes that possess promoters with higher levels of DNA methylation are likely to avoid miRNA regulation (Figure 1). 

This report provides the first attempt to uncover such an important and complex regulation system. Unlike many previous exploratory studies, focused on delineating the effects of a single epigenetic mechanism on gene expression, this study aimed at understanding how DNA methylation and miRNA reciprocally regulate the expression of target genes at the genome level. Although the results obtained suggest a complementary relationship between DNA methylation and miRNA regulation, the way in which the two mechanisms cooperate remains poorly understood.

An integrated genome-wide analysis of DNA methylation (methylome distribution), miRNAome and mRNA transcripts in cattle skeletal muscle development has been carried out in order to investigate the coordinated action of several epigenetic modifications [72]. This research, unlike the previous ones, not only examined methylation in promoter regions, but also in gene bodies. As expected, a negative correlation between core promoter methylation and gene expression has been found in both fetal and adult bovine skeletal muscle. In fact, promoters of highly-expressed genes exhibited low methylation levels, as in myosin light chain 2 (MYL2) or dystrobrevin binding protein (DTNBP1). Instead, the promoters of lowly-expressed genes were usually highly methylated, as in the case of cellular retinoic acid binding protein 2 (CRABP2) or laminin B1 (LAMB1). DNA methylation level was higher in the adult bovine stage compared to the fetal period, and the differences in methylation degree might have partially contributed to the progression through the different stages of muscle development. Among the genes of interest were CRABP2, a key modulator of skeletal muscle differentiation, and MYL2, a well-known gene related to the biosynthesis of myosin. While gene body DNA methylation positively correlates with gene expression, its functional role is still unknown and does not allow drawing any causal conclusion. It is worth noting that, contrary to the previous study, the average methylation levels were higher for the miRNA-targeted genes than for the non-miRNA-targeted ones, in both fetal and adult bovine libraries. These results suggest that miRNA activity on target genes may somehow encourage methylation of the gene, or, that it is so important to repress certain genes during development that both DNA methylation and miRNAs are in place to fully switch their expression off. By using the integrated approach between analyses of methylome and miRNAs, this study has also confirmed the importance of some miRNAs, such as miR-1, miR-133, and miR-206, in targeting multiple genes related to muscle development. The aim of this study not only was to confirm or identify new possible muscle development-related genes, but also to highlight the combined action of DNA methylation and miRNA post-transcriptional regulation which have, so far, been investigated individually or analyzed focusing on restricted target genes in muscle development.

Epigenetics fine-tunes gene expression in response to extracellular stimuli or pathological states. Several reports have described dynamic changes in DNA methylation pattern and miRNA expression in diseases, which may contribute to the pathogenic progression and, therefore, be targeted by new therapeutic strategies. Increasing evidence has pointed to miRNAs role in the post-transcriptional regulation of gene expression in skeletal muscle response to exercise [73,74,75]. Furthermore, both acute and chronic exercises have a significant impact on DNA methylation, in a tissue- and gene-specific manner in humans [76]. A comprehensive analysis of these epigenetic mechanisms has been conducted in a recent study on skeletal muscle of type 2 diabetes mellitus (T2D) patients, following chronic exercise training [77]. Microarray analyses were performed on skeletal muscle of obese Polynesian patients with T2D, before and after 16 weeks of endurance or resistance exercise, generating epigenomic and transcriptomic networks. Modulation of DNA methylation and miRNA expression was higher after endurance training and was related with metabolic and microvascular plasticity, important to diabetes rehabilitation. Both resistance and endurance training induced hypomethylation of the DNA, despite affecting the genes involved in different molecular pathways. Namely, in response to endurance training, differential methylation mostly affected the genes related to lipid and carbohydrate metabolism, metabolic diseases, cell death, and survival. On the contrary, the top-ranked functional networks and categories responding to resistance training were cellular assembly and organization, cellular development, tissue morphology and cardiovascular system development and function. At the same time, endurance and resistance training induced changes in the expression of 25 and 23 miRNAs, respectively. The analysis of the predicted targets suggests that miRNAs regulate the genes involved in the regulation of transcription, lipid and glucose metabolism, and myofibril and connective tissue development in response to endurance training. The targets involved in controlling gene expression and blood vessel development were influenced by resistance training. The authors concluded that extensive metabolic and molecular reprogramming are more pronounced in response to chronic endurance training than in the resistance one, which indicates greater efficiency in diabetes rehabilitation. Integrating transcriptome and methylome analyses supported the hypothesis that DNA methylation and miRNA expression cooperate in the metabolic plasticity of skeletal muscle in response to endurance training. In particular, the metabolic reprogramming, evinced by the transcriptome analysis, was connected to epigenetic regulation by downregulated miR-29a and an overrepresented metabolic methylome [77].

## 3. MicroRNAs and Histone Modifications

Various miRNAs control the chromatin structure by affecting the “histone code” and targeting key enzymes, known as histone modifiers. Furthermore, histone modifications are directly involved in the regulation of cell-specific expression of miRNAs and modulate their levels in various physiological and pathological conditions [44,45]. The connection between miRNAs and histone modifications has undoubtedly added a new layer of regulation to the already-existing knowledge about modulation of cellular processes.

### 3.1. MicroRNAs and Histone Acetylation/Deacetylation

**Figure 2:**
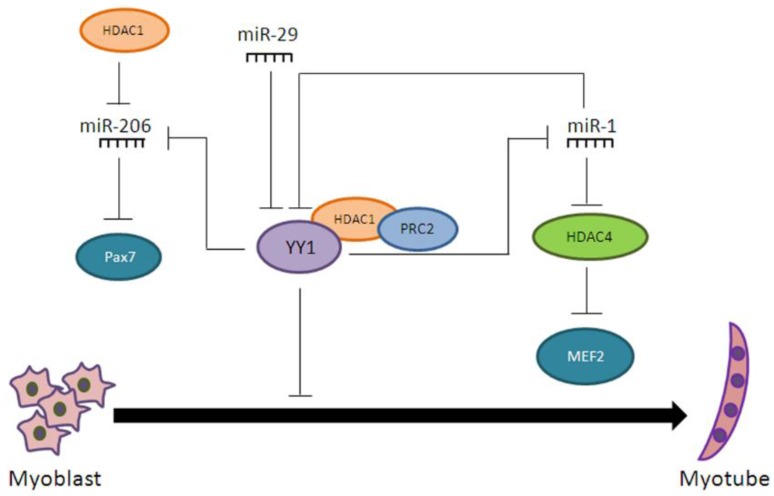
Coordinated actions of microRNAs with chromatin remodeling factors in myogenesis. YY1 represses myogenesis in the presence of HDAC1 and PRC2, also by inhibiting the expression of several miRNAs. These, in turn, inhibit YY1 expression.

The interplay between miRNAs and HATs/HDACs, as well as the coordinated actions of the two epigenetic mechanisms, play a paramount role in myogenesis, especially during muscle development. One of the miRNAs involved in myogenesis is miR-1, which promotes muscle regeneration also by targeting HDAC4 [78] that, in turn, inhibits the expression of the myogenic factor Myocyte enhancer factor 2 (MEF2) [79]. Similarly, miR-29 promotes myogenesis through the inhibition of the transcriptional regulator Ying Yang 1 (YY1), which, in conjunction with the epigenetic factors PRC2 and HDAC1, acts as a repressor of muscle-specific gene expression [80]. Interestingly, YY1 is also a target of miR-1 and represses the expression of miR-1, miR-133, and miR-206 in myoblast, by recruiting PRC2 on their promoters [81]. miR-206 promotes myoblast differentiation by repressing Pax7 [78], and its expression is strictly controlled by HDAC1 during myogenesis [82]. Thus, miRNAs regulate the expression of HDACs and other chromatin remodeling factors, creating regulatory circuitries that supervise and reinforce the epigenetic regulation of gene expression in myogenesis (Figure 2).

Changes in epigenetic regulation are often associated with diseases. It has been reported that the expression of miR-449a is significantly downregulated in skeletal muscle of diabetic mice [83]. miR-449a is an intronic miRNA co-transcribed with its host gene, Cdc20b. Many histone acetylation marks were found on the Cdc20b promoter, suggesting that HATs and HDACs may affect its expression. Interestingly, skeletal muscle of diabetic mice showed increased HDAC expression and activity, which inversely correlate with miR-449a expression, indicating a connection between the two epigenetic mechanisms. In fact, treatment of diabetic mice with an HDAC inhibitors (HDACi) significantly increased miR-449a levels in skeletal muscle. Therefore, HDAC inhibition, already identified as a therapeutic strategy for diabetes, could improve skeletal muscle health in diabetes by restoring miR-449a expression to control levels. 

The potential of HDACi as a treatment for Duchenne Muscular Dystrophy has been exploited in mdx mice [84]. In the absence of dystrophin, reduced levels of intracellular nitric oxide [85] lead to hyper-activation of HDAC2 [86]. HDAC2 targets, such as miR-1 and miR-29, are relevant to the progression of muscular dystrophy, regulating cellular metabolism and fibrosis [82]. Moreover, HDACi promotes the myogenic program in fibro-adipogenic progenitors (FAPs), while suppressing the fibro-adipogenic phenotype in dystrophic muscles [87]. In particular, HDAC inhibition leads to the upregulation of the expression of miR-1/2, miR-133, and miR-206 in FAPs. These muscle-specific miRNAs target a definite subunit of the chromatin remodeling complex SWI/SNF, promoting promyogenic differentiation [87].

The role of HDAC4, and its regulator miR-206 in compensatory reinnervation of skeletal muscle and disease progression, have been described in a mouse model of amyotrophic lateral sclerosis (ALS) [88]. miR-206 was shown to have a protective role in ALS by promoting reinnervation and slowing down ALS progression in mice. miR-206 exerts its action by repressing the expression of HDAC4 at the neuromuscular junctions (NMJs), leading to the consequent upregulation of the fibroblast growth factor binding protein 1. The latter encodes a secreted factor that interacts with, and strengthens, the bioactivity of fibroblast growth factor members, crucial for NMJ innervations [88]. This is another extraordinary example of how a miRNA fine-tunes the expression of a chromatin-remodeling enzyme in a restricted location within skeletal muscle, following denervation. Indeed, the expression of HDAC4 was significantly upregulated in skeletal muscle following denervation [89] and in ALS patients [90]. Moreover, HDAC4 expression in skeletal muscle positively correlated with the ALS progression rate and inversely correlated with the extent of reinnervation [90], confirming the negative role of muscle HDAC4 in the reinnervation process in ALS patients.

### 3.2. MicroRNAs and Histone Methylation

**Figure 3:**
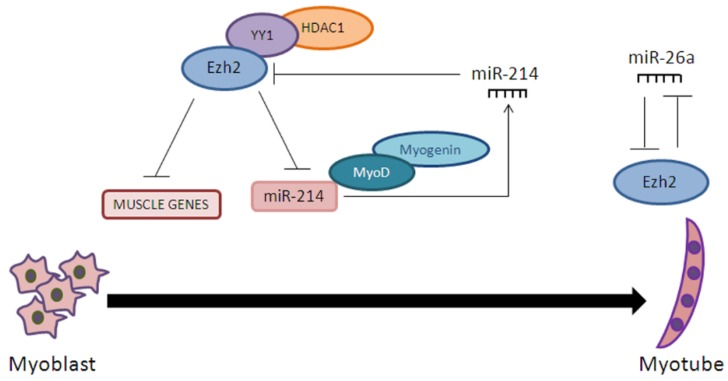
Ezh2—miRNA feedback loop in myogenesis. In undifferentiated myoblasts, Ezh2 represses miR-214, miR-26a, and myogenic gene expression. Upon differentiation, miR-214 and miR-26a are produced and Ezh2 expression is repressed, allowing myogenic differentiation to occur.

The role of microRNAs and histone methylation during myogenesis has been extensively studied in recent years. Various miRNAs modulate chromatin structure and gene transcription by regulating histone methylation. In a study in which the expression of miRNAs from proliferating myoblasts to terminally-differentiated myotubes was analyzed, miR-26a was identified as upregulated during myogenesis [79]. By using a bioinformatics approach, a subunit of PRC2 complex, Ezh2, was identified as a possible miR-26a target gene. Coherently, the expression of miR-26a and Ezh2 inversely correlated during muscle differentiation. Prior to myogenesis, the histone methyltransferase Ezh2, along with YY1 and HDAC1, bound to the E-box regions of muscle-specific gene promoters, causing their silencing primarily through H3K27 three-methylation. Upon activation of myogenesis, the Ezh2-containing complex disassociated from chromatin, maintaining the accessibility of the promoter regions to transcriptional activators. Therefore, an increased level of miR-26a during myogenesis serves to post-transcriptionally repress Ezh2 so that it is no longer able to elicit its suppressive effects on myogenesis [91] (Figure 3). However, this study does not clarify the mechanism completely. It is still unclear whether miR-26a-mediated Ezh2 mRNA suppression causes the decrease in Ezh2 protein expression observed during myogenesis or whether the decline in Ezh2 protein acts as a negative feedback, causing the decrease in Ezh2 mRNA expression. In addition to miR-26a, the Ezh2 3′ UTR is also targeted by miR-214 [92]. It is likely that miR-214 and miR-26a repress Ezh2 at distinct developmental steps. Both miR26a and miR-214 affect Ezh2 and, in turn, they are modulated by Ezh2 in a negative feedback loop. While miR-26a may be relevant at later stages of differentiation, as its expression occurs in terminally-differentiated muscle cells, miR-214 accumulation is observed at the very initial stages of cell differentiation. A working model in which the downregulation of Ezh2, taking place at the initial phase of muscle differentiation, together with MyoD and/or myogenin recruitment, promotes the expression of miR-214 has been proposed (Figure 3). While in myoblasts, PRC2 binds and represses miR-214 transcription, during differentiation, PRC2 is disengaged and MyoD/myogenin are recruited at the miR-214 promoter region, promoting its transcription. After being transcribed, miR-214 loops back to target the Ezh2 3′ UTR, thus reducing Ezh2 protein accumulation. 

Thus, miR-214 can impact the transcription controlled by PRC2, by regulating Ezh2 protein levels, introducing an additional level of PRC2 regulation via a post-transcriptional mechanism. This network motif, named the two-node bistable feedback loop [93], ensures that the system is robust enough to effectively and rapidly reduce Ezh2 availability at critical stages as those regulating skeletal muscle cell differentiation.

Another study highlighted the connection between the arginine methyltransferases (Prmts) and the myogenic miRNA expression [94]. Prmt5 is required for myogenin transcription, therefore, it is indirectly indispensable for the expression of myogenic miRNAs (miR-1-1/2 and miR-133a-1/2) [95]. By contrast, Prmt4 directly binds to, and modifies, histones in myogenic miRNA regulatory sequences, and is the requisite for the binding of both SWI/SNF and myogenin transcription factors. Moreover, Prmt4 binds to, and is required, for the expression of genes at later stages of skeletal muscle differentiation [95,96]. This is another example of how histone-modifying enzymes and miRNAs cooperate during myogenesis.

## 4. Conclusions

There has been growing interest in epigenetic mechanisms in recent years, highlighting the importance of epigenetics in regulating developmental signaling, as well as cellular adaptation to external stimuli or diseases. In the last decade, the study of miRNA biology has attracted remarkable attention, resulting in rapid advances. The increasing literature exploring the role of miRNAs has clarified their biological functions and involvement in pathological states, suggesting that miRNAs may be used as targets for therapeutic approaches or biomarkers for diagnosis. By studying epigenetic mechanisms, cooperated actions between miRNAs and other epigenetic factors have emerged, depicting a more complex and sophisticated layer of gene regulation. Thanks to the advances in new generation sequencing, integrated multi-omic analyses have started to define the interaction between miRNAs and epigenetic factors, discovering coordinated circuitries that modulate gene expression during myogenesis and in disease. These studies will facilitate the development of novel, combined approaches to prevent or treat skeletal muscle in several disease conditions.
